# hASC and DFAT, Multipotent Stem Cells for Regenerative Medicine: A Comparison of Their Potential Differentiation In Vitro

**DOI:** 10.3390/ijms18122699

**Published:** 2017-12-13

**Authors:** Marco Saler, Laura Caliogna, Laura Botta, Francesco Benazzo, Federica Riva, Giulia Gastaldi

**Affiliations:** 1 Department Clinical Surgical, Diagnostic and Pediatric Sciences, Plastic and Reconstructive Surgery Unit, University of Pavia, 27100 Pavia, Italy; 2 Orthopedics and Traumatology Clinic, IRCCS Policlinico San Matteo Foundation, 27100 Pavia, Italy; l.caliogna@smatteo.pv.it (L.C.); f.benazzo@smatteo.pv.it (F.B.); 3 Department of Biology and Biotechnology “Lazzaro Spallanzani”, University of Pavia, 27100 Pavia, Italy; laura.botta@unipv.it; 4 Department of Clinical Surgical, Diagnostic and Pediatric Sciences, Locomotor System Diseases Unit, University of Pavia, 27100 Pavia, Italy; 5 Centre for Health Technologies, University of Pavia, 27100 Pavia, Italy; federica.riva01@unipv.it (F.R.); giulia.gastaldi@unipv.it (G.G.); 6 Department of Public Health, Experimental and Forensic Medicine, Histology and Embryology Unit, University of Pavia, 27100 Pavia, Italy; 7 Department of Molecular Medicine, Human Physiology Unit, University of Pavia, 27100 Pavia, Italy

**Keywords:** adipocytes, adipose tissue, cell culture in vitro, dedifferentiated fat adipose tissue, fibroblast-like, human adipose stem cells

## Abstract

Adipose tissue comprises both adipose and non-adipose cells such as mesenchymal stem cells. These cells show a surface antigenic profile similar to that of bone-marrow-derived MSC. The cells derived from the dedifferentiation of mature adipocytes (DFAT) are another cell population with characteristics of stemness. The aim of this study is to provide evidence of the stemness, proliferation, and differentiation of human adipose stem cells (hASC) and DFAT obtained from human subcutaneous AT and evaluate their potential use in regenerative medicine. Cell populations were studied by histochemical and molecular biology techniques. Both hASC and DFAT were positive for MSC markers. Their proliferative capacity was similar and both populations were able to differentiate into osteogenic, chondrogenic, and adipogenic lineages. DFAT were able to accumulate lipids and their lipoprotein lipase and *adiponectin* gene expression were high. Alkaline phosphatase and *RUNX2* gene expression were greater in hASC than in DFAT at 14 days but became similar after three weeks. Both cell populations were able to differentiate into chondrocytes, showing positive staining with Alcian Blue and gene expression of *SOX9* and *ACAN*. In conclusion, both hASC and DFAT populations derived from AT have a high differentiation capacity and thus may have applications in regenerative medicine.

## 1. Introduction

Multipotent mesenchymal cells (MSCs) are adult stem cells that can be isolated from various tissues and expanded in vitro; by virtue of their pluripotency, MSCs are a source for clinical applications, mainly tissue injury and immune disorders [1]. In 2006 the International Society for Cellular Therapy (ISCT) proposed criteria to define the identity of these cells [2].

MSCs are extracted from different tissues—bone marrow (BM-MSCs) [3], fetal membranes of term placenta (hFMSCs) [4,5], dental pulp (hDMSCs) [6], ovarian follicular liquid (FL) [7,8], and adipose tissue (hASCs) [9,10,11]—by different isolation protocols. These cells can be easily maintained or expanded in vitro and have the ability to assume osteogenic [12], adipogenic, and chondrogenic, as well as neurogenic [13] phenotypes. The differentiation of MSC to endothelial cells is still unproven [14,15]. Adipose tissue (AT) contains a greater number of stem cells compared to other tissues [16] and the cells can be harvested with a minimally invasive surgical procedure.

AT comprises several cell types including preadipocytes, endothelial, and immune cells, but the main type are mature adipocytes [17]. These cells have a typical morphology characterized by the presence of a single and large cytoplasmic lipid droplet that occupies ~90% of the cell volume [18]. In the past, researchers thought the process of adipocyte differentiation was irreversible, but recent studies in mice suggest that mature adipocytes are capable of changing their phenotype under physiological stimuli, thus acquiring morphology and physiology [16,19].

Recent in vitro studies have shown that mature adipocytes isolated from AT can be reprogrammed into multipotent stem cells [20]. These adipocyte-derived cell lines, which are obtained through the “ceiling culture” method, are generally defined as dedifferentiated fat cells (DFAT). DFAT cells show a fibroblast-like morphology and have the characteristics of precursor cells with multilineage potential [21]. DFAT have been obtained from mouse, rat, porcine, rabbit, bovine, feline, and human sources [20,22,23]. DFAT have an active proliferation ability and can differentiate into osteoblasts [24] or chondrocytes [25], or can differentiate back into mature adipocytes [26] under appropriate culture conditions. Compared with ASCs and other adult stem cells, DFAT cells have several advantages such as abundance, isolation method, homogeneity, and a low immunogenicity after transplantation [22,27,28].

In the present study, we compare the cell-surface antigen expression, proliferative capacity, and multilineage differentiation ability of hASCs and DFAT populations. The innovative aspect of this research is the analysis of two different multipotent cells lines, both obtained from the adipose tissue of the same patient.

## 2. Results

### 2.1. Dedifferentiated Mature Adipocyte (DFAT) and Human Adipose Stem Cell (hASC) Cultures

**Figure 1:**
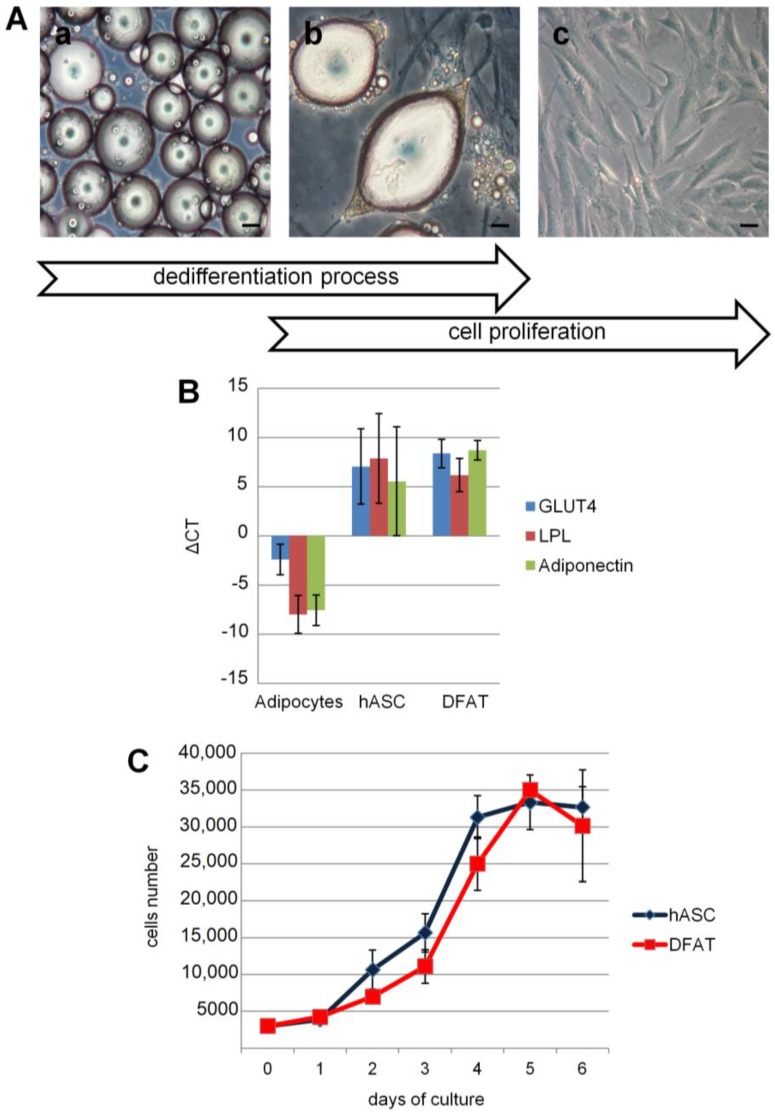
Dedifferentiation process and culture of mature adipocytes. (**A**) At day 0, mature adipocytes flattened to the upper surface of the flask (**a**). During the seven day-culture period, cells underwent a differentiation process: they lost the lipid content (**b**) and attached to the flask by converting to fibroblast-like fat cells (DFAT). DFAT were cultured following a conventional method (**c**). Bars, 100 µm; (**B**) phenotypic analysis of human mature adipocytes, human adipose stem cells (hASC) and DFAT at first passage: *lipoprotein lipase* (*LPL*), *GLUT4* and *adiponectin* expression in DFAT and in hASC were about 10^4^ times lower than mature adipocytes. Gene expression was normalized using GUSB as housekeeping gene (ΔCT); (**C**) curves of viability: the growth kinetics of DFAT cells was similar to that of hASC during week 1.

hASC cells were seeded in a flask and adhered to the surface, and showed fibroblast-like morphology under light microscopy. Mature adipocytes with a large lipid droplet and spherical shape were seeded in flasks and maintained in a “ceiling culture”. During a seven-day period, the mature adipocytes began a dedifferentiation process characterized by a loss of lipid content and a change from a spherical to a fibroblast-like morphology (Figure 1Aa–c).

### 2.2. Expression of Cell-Surface Antigens by DFAT and hASC

The cell surface antigen profile of DFAT was analyzed and compared with the profiles of hASC at passage 0. DFAT cells were uniformly positive for CD13 (aminopeptidase N), CD73 (5′-nucleotidase), CD90 (Thy-I), and CD105 (endoglin), but negative for CD14 (myelomonocytic differentiation antigen); less than 1% of these cells expressed CD34 (hematopoietic progenitor cell antigen) and CD45 (protein tyrosine phosphatase, receptor type C). This profile was similar to previous findings for BM-MSC and umbilical vein stem cells (UVSCs). The surface antigen profile of hASC at passage 0 was essentially the same as that of DFAT cells (Table 1).

### 2.3. Dedifferentiation Analysis by Real-Time RT-PCR

To evaluate changes in the gene expression profile during the dedifferentiation process, the expression of several markers in DFAT and hASC cells compared with mature adipocytes was analyzed using real-time RT-PCR. Abundant expression of mature adipocytes markers such as *adiponectin, GLUT4* and *LPL* were revealed, whereas these markers were reduced about 10^4^-fold in both DFAT and hASC cells (Figure 1B). These results showed that DFAT cells lose the characteristics of mature adipocytes but acquire the specific phenotype of MSC.

### 2.4. Generation Time, Viability, and Proliferation Capacity

Cell generation time, viability, and proliferation capacity increased in both DFAT and hASC. Both DFAT and hASC showed an exponential increase without any statistically significant difference (Figure 1C). 

Proliferative capacity expressed as generation time was 1.65 ± 0.09 and 1.588 ± 0.07 in DFAT and in hASC respectively, showing similar kinetics in both cell populations. No gender-related differences were observed.

### 2.5. Evaluation of Differentiation

#### 2.5.1. Adipogenic Differentiation

**Figure 2:**
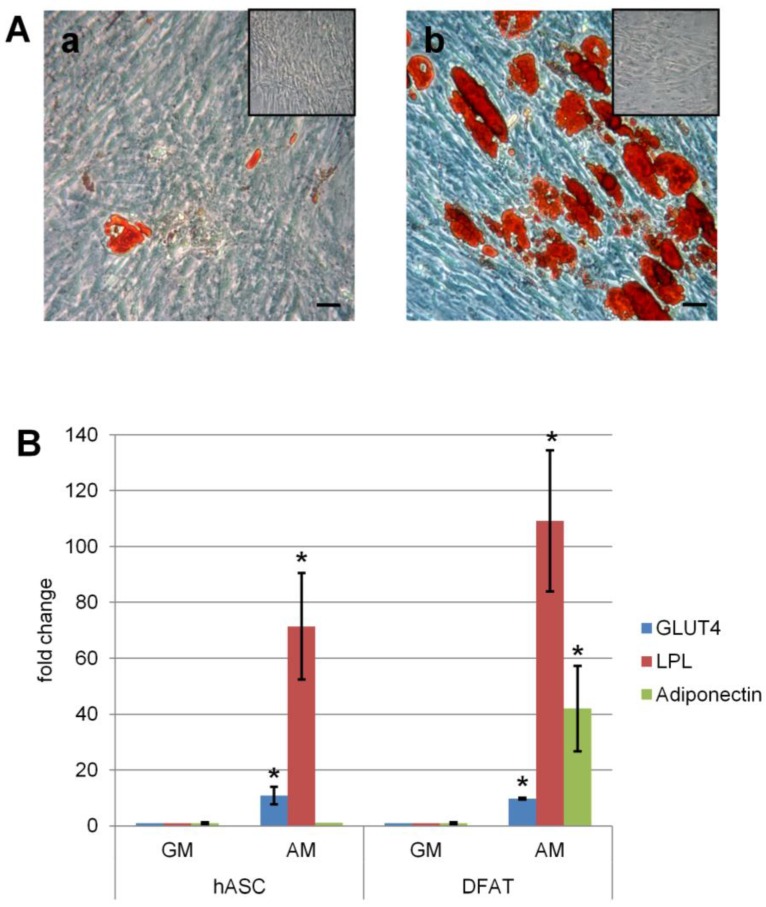
Adipogenic differentiation in hASC and DFAT in the presence (AM: adipogenic medium) or absence (GM: growth medium) of adipogenic factors. (**A**) Microscopic evaluation in hASC (**a**) and DFAT (**b**) cells of the presence of intracellular lipid droplets by Oil Red O stain at 15 days and their respective control (inset). Bars, 100 µm; (**B**) expression of *LPL*, *GLUT4* and *adiponectin* of DFAT and hASC cells after 15 days in culture. Data expressed as fold change in cell in adipogenic medium (AM) versus cell in growth medium (GM). * *p* ≤ 0.05.

Adipogenic differentiation of both hASC and DFAT was evaluated after 15 days in adipogenic medium. Gene expression analysis revealed that the fold change of *adiponectin* and *lipoprotein lipase* (*LPL*), functional markers of adipose tissue cells, was higher in DFAT than in hASC. *GLUT4* fold change was similar in the two populations (Figure 2B). The amount of lipid droplets accumulated was determined by Oil Red O staining. After seven days of cell culture in adipogenic medium, both hASC and DFAT showed the presence of small intracellular lipid droplets. However, at 15 days the accumulation of lipid droplets in hASC (Figure 2Aa) was less than in DFAT (Figure 2Ab); the control samples maintained in growth medium were not positive to the staining, confirming their undifferentiated state. Densitometric analysis and quantification of lipid droplets at 15 days showed that DFAT accumulated a statistically significant higher amount of lipid droplets than hASC (Table 2).

#### 2.5.2. Osteogenic Differentiation

**Figure 3:**
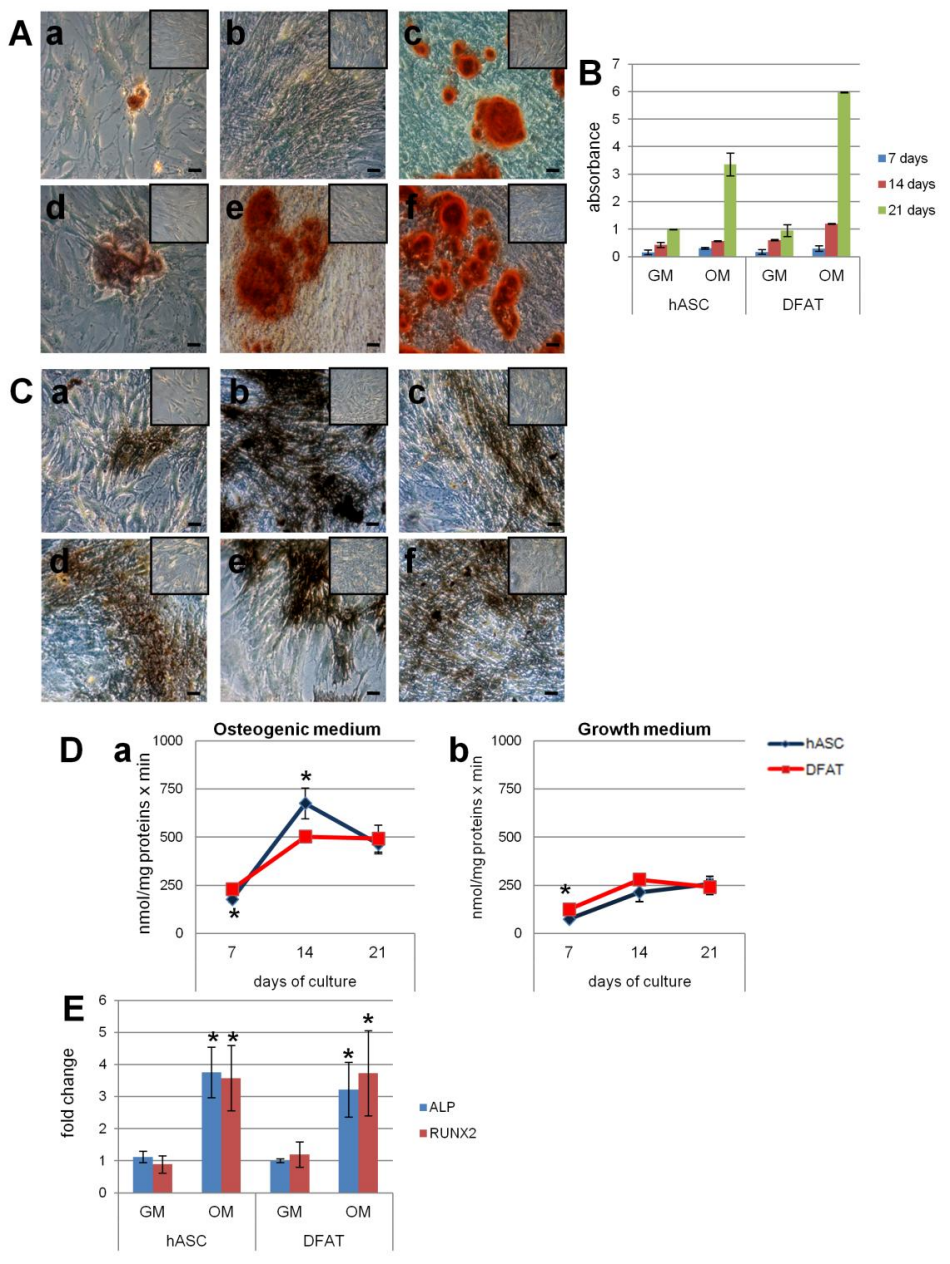
Osteogenic differentiation in hASC and DFAT in the presence (OM: osteogenic medium) or absence (GM: growth medium) of osteogenic factors. (**A**) Microscopic evaluation of calcium deposits in hASC (**a**–**c**) and DFAT (**d**–**f**) by Alizarin Red staining at 7 (**a**,**d**), 14 (**b**,**e**) and 21 (**c**,**f**) days in culture and their respective control (inset). Bars, 100 µm; (**B**) spectrophotometric quantification of Alizarin Red staining of calcium deposits in hASC and DFAT at seven, 14, and 21 days in culture and their respective control; (**C**) microscopic evaluation of the presence of ALP in hASC (**a**–**c**) and DFAT (**d**–**f**) and their respective control (inset) by histoenzymatic stain at seven (**a**,**d**), 14 (**b**,**e**), and 21 (**c**,**f**) days in culture. Bars, 100 µm; (**D**) ALP activity assay at seven, 14, and 21 days in osteogenic medium (**a**) and growth medium (control) (**b**). * *p* ≤ 0.05 vs. DFAT control at seven days, one-way ANOVA method followed by Newman-Keuls’ *Q* test (GraphPad Prism 4.00, 2003); (**E**) expression of *ALP* and *RUNX2* in DFAT and hASC cells at 21 days of culture in osteogenic (OM) and growth (GM) medium (control). The data were expressed as fold change versus controls. * *p* ≤ 0.05 vs. control.

After 21 days, the osteogenic differentiation of hASC and DFAT was evaluated by gene expression of ALP, a protein associated with the bone formation, and RUNX2, a key transcription factor associated with osteoblast differentiation. In both populations, the expression of both genes by real time RT-PCR in the osteogenic medium was statistically higher than in the growth medium (Figure 3E).

The mineralization process was determined by Alizarin Red S staining showing a red color on crystallized calcium salt under light microscopy. After seven, 14, and 21 days of cell culture in osteogenic medium, calcium deposits increased in both hASC and DFAT. Calcium deposition capacity appeared to be more intense in DFAT than in hASC and was absent in the controls (Figure 3A).

After the dye bound to calcium deposits was solubilized with cetylpyridinium chloride (CPC), spectrophotometric analysis confirmed a progressive increase in mineralization at all three endpoints in both populations. In particular, at day 21 of the differentiation process, bound Alizarin Red in hASC and in DFAT was 3.5 times and 6 times greater, respectively, than in control cells (Figure 3B).

The presence of ALP, a key enzyme in the mineralization process, was evidenced in the cells by a histoenzymatic reaction that yielded a brown staining. After seven, 14, and 21 days in osteogenic medium, ALP positive cells increased in both hASC and in DFAT (Figure 3Ca–f). At 14 days, the ALP positive cells in the DFAT (Figure 3Ce) were lower than in the hASC population (Figure 3Cb). The control cells cultured in growth medium showed a non-significant increase in *ALP* compared to the differentiated cells.

The ALP activity was evaluated at seven, 14, and 21 days by quantifying p-nitrophenol product. ALP activity in both the hASC and DFAT populations maintained in osteogenic medium was significantly higher than in the controls; in particular at day 14, ALP activity was lower in DFAT than in hASC but by day 21 the difference had disappeared (Figure 3D).

#### 2.5.3. Chondrogenic Differentiation

**Figure 4:**
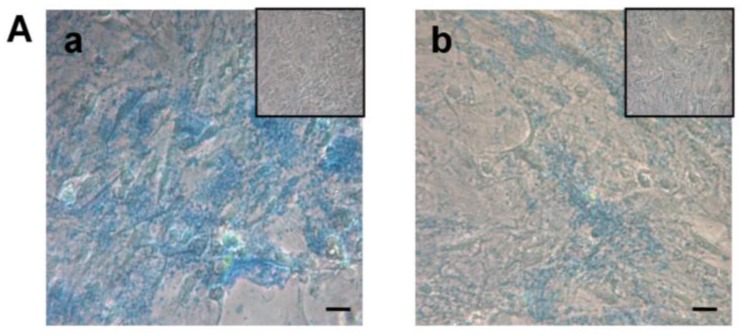

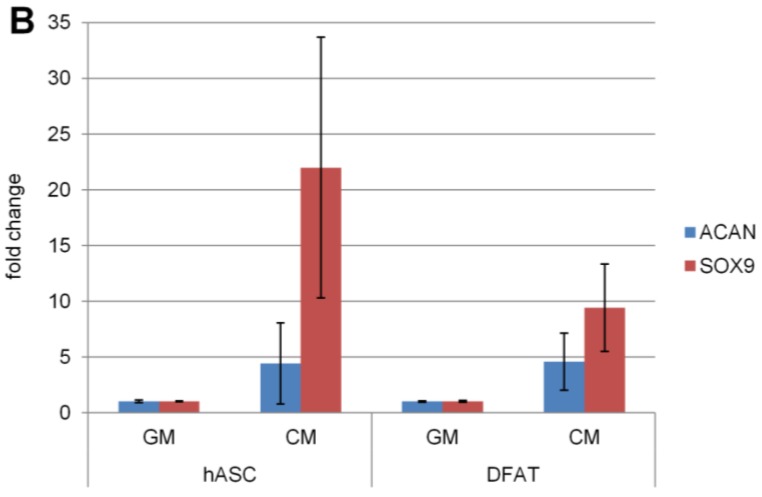
Chondrogenic differentiation in hASC and DFAT in the presence (CM: chondrogenic medium) or absence (GM: growth medium) of chondrogenic factors. (**A**) Microscopic evaluation of acid mucopolysaccharides in hASC (**a**) and DFAT (**b**) cells and their respective control (inset) by Alcian Blue 8GX stain at 30 days. Bars, 100 µm; (**B**) expression of *ACAN* and *SOX9* of DFAT and hASC cells after 30 days in culture. Data are expressed as fold change of cells in a chondrogenic medium versus a growth medium.

The chondrogenic differentiation of both hASC and DFAT was evaluated at 30 days by gene expression of ACAN, a gene encoding a cartilage-specific proteoglycan core proteins and SOX9, a transcription factor that regulates successive steps of chondrocyte differentiation *ACAN* and *SOX9* expressions did not show any statistically significant difference from the control samples in GM (Figure 4B).

Cell proteoglycans were evaluated by the Alcian Blue stain, which binds to the chondroitin-sulfate and keratan-sulfate synthesized and secreted by differentiated cells. After 30 days in chondrogenic medium, both hASC and DFAT showed an increase in these proteoglycans both at the intra and extracellular level. Under light microscopy, the DFAT cells showed a lower differentiation capacity than the hASC. The cells maintained in a growth medium were negative to the dye (Figure 4A).

## 3. Discussion

Following trauma, cancer, and malformations, the loss of bone tissue must be repaired. Appropriate therapies include the transplantation of autologous or allograft bone or the replacement of a prosthetic structure. A new frontier is represented by human regenerative medicine and tissue engineering. In this regard, the novel use of living cells and biomaterials can afford a significantly better alternative to artificial implants. The choice of cells is pivotal: these cells should be easily obtainable in an adequate amount, should adhere and proliferate on biomaterials, and then should be able to regenerate the damaged tissue. Subcutaneous adipose tissue comprises mature adipocytes and many other cell types: endothelial cells, pericytes, adipose precursor cells, and fibroblast-like cells. This tissue contains cells that express specific markers of mesenchymal stem cells, termed hASC. Literature data about the expression of CD34 in hASC are conflicting: it is positive according to some studies [29,30] and negative according to others [31,32]. In particular, it has been suggested that some hematopoietic cells (CD34+) are present in earlier passages and lost in the next steps. hASCs are positive for mesenchymal stem cell markers as CD13, CD73, CD90, and CD105, and negative for CD14, CD34 [31,32], and CD45, according to Tocci et al. (2003) [33]. The cells obtained from mature adipocytes and maintained in “ceiling mode” start a gradual process of dedifferentiation, highlighted by positive markers of mesenchymal stem cells [28,34]. This dedifferentiation process was also observed in other cells (i.e., chondrocytes and smooth muscle cells) and seems to be induced by the hypoxia that occurs during the “ceiling cultures” [35,36,37]. The mechanisms that induce the dedifferentiation process of mature fat cells and the specific surface markers of DFAT populations must be studied more thoroughly. In this study, the two cell populations isolated from the same patient showed characteristics typical of MSC. hASCs and DFAT in vitro showed similar proliferative capacity (expressed as the generation time obtained from the respective growth curves). By adding appropriate factors to the culture medium DFAT, hASC and BM-MSC cells can be induced to differentiate into osteoblastic, adipogenetic, and chondroblastic lineages [37,38,39]. All the evaluated differentiation parameters were positive for the presence of acidic mucopolysaccharides: alkaline phosphatase, calcium deposits, and the intracytoplasmic accumulation of lipid droplets. Moreover, the specific genes of the osteoblastic, adipogenic, and chondroblastic lines were expressed in DFAT. These data suggest that DFAT acquire stem cell characteristics and therefore they may be a viable substitute for stem cells in cell therapies and tissue engineering. Further, DFAT can be obtained easily and in large amounts with a minimally invasive harvesting method that avoids the use of the viral vectors necessary for cellular reprogramming (iPS); it is obtained from the same patient to be treated, thus avoiding immune responses. The comparison between the two cell populations obtained from the subcutaneous adipose tissue of the same patient suggests that there are some differences in their differentiation capacity. The DFAT responded better to the addition of the adipogenic medium than the hASC: the accumulation of intracellular lipid droplets was observed after only seven days of culture, while for the hASC at least 15 days were required; these data were further confirmed by quantification of lipidic droplets at 15 days. Also, after 15 days the gene expression of *LPL* and *adiponectin* was higher in DFAT than in hASC. Presumably the cells derived from the dedifferentiation of mature adipocytes were already “committed” to the adipocyte lineage, therefore more suitable for tissue engineering of the adipose tissue. Indeed, a different degree of differentiation has been shown for MSCs according to their site of origin [40]. In the salamander *Ambystoma mexicanum*, the phenomenon of limb regeneration takes place without complete dedifferentiation to a pluripotent state, starting from the blastema, which is a heterogeneous collection of restricted progenitor cells [41]. In humans the wound repair process requires a non-functioning mass of fibrotic tissue: no blastema has been recognized but MSCs are able to start the repair process.

The osteoblastic differentiation capacity of DFAT and hASC seem to be similar, although there were some small differences: ALP activity evaluated with biochemical and histoenzymatic methods was greater in hASC than in DFAT at 14 days of differentiation, while at 21 days it was similar. Instead, the gene expression of *ALP* and *RUNX2* at 21 days seemed to be higher in DFAT than in hASC. The monolayer culture in chondrogenic medium induced the chondrogenic differentiation of both hASC and DFAT, even if stem cells preferentially differentiated into the chondrogenic line when maintained in pellet culture or seeded on alginate three-dimensional structures as scaffolds [42]. After 30 days of differentiation, the hASC apparently produced a greater amount of acidic mucopolysaccharides than the DFAT; there were no differences in the gene expression of *ACAN* between the two cellular populations. *SOX9* expression after 30 days of differentiation was lower in DFAT than hASC. This finding was in agreement with previous reports that showed a peak of expression of *SOX9* in the cells after 14 days of culture, followed by a drastic drop at the third week [28]. 

## 4. Materials and Methods

### 4.1. Human Adipose Tissue Specimen Collection

Subcutaneous AT was obtained from anatomical specimens harvested during surgery for hip prosthesis implantation from the peritrocanteric region of 12 healthy patients (six males and six females). The age range was 60–70 years and the body mass index was 22.5–26.5. The specimens were sampled by a surgeon in ~3 × 3 cm segments and preserved in sterile containers filled with saline solution enriched with 1% penicillin (100 U/mL) and streptomycin (100 µg/mL) (Eurobio, Courtaboeuf, France), and transported to the laboratory for processing. The study was conducted in accordance with the 1975 Declaration of Helsinki; informed consent was obtained from all patients before surgery and the protocol was approved by Ethics Committee of San Matteo Foundation, Research and Care Institute, Pavia, Italy (P-20100022172, 11 March 2013).

### 4.2. Isolation of Stem Cells from Adipose Tissue (hASCs)

Adipose tissue samples were divided in two parts, finely fragmented and incubated with Dulbecco’s Modified Eagle’s Medium F12 HAM (DMEM F12-HAM) (Sigma-Aldrich, St. Louis, MO, USA) supplemented with 10% fetal bovine serum (FBS) (Biowest, Nuaillé, France), 100 U/mL penicillin, 100 µg/mL streptomycin, 0.25 µg/mL amphotericin (growth medium: GM) (all from Eurobio) and 0.01% collagenase type II (Sigma-Aldrich) at 37 °C in a shaking water bath for 1 h. After incubation and neutralization of collagenase solution with an equal volume of DMEM, the cell suspension was filtered (membrane pore size: 100 µm) (Merck Millipore, Darmstadt, Germany) to remove the debris in the digested tissue and centrifuged at 1200 rpm for 10 min at 4 °C. The pellet was suspended in GM, centrifuged twice, resuspended in the same medium, and treated with lysis solution (0.15 M NH_4_Cl, 10 mM KHCO_3_, 0.1 mM Na_2_-EDTA, pH 7.22) for 10 min at 4 °C. After the addition of GM, the suspension was centrifuged and the resulting pellet containing hASC was suspended in GM. The hASCs were seeded in flasks (Greiner Bio-One GmbH, Frickenhausen, Germany) and cultured in GM up to 95% confluence in a humidified atmosphere of 95% air with 5% CO_2_ at 37 °C. The adherent cells were trypsinized with Trypsin EDTA (Biowest, Nuaillé, France), and 1 × 10^5^ hASCs/cm^2^ tissue culture plate were seeded in new flasks. These passages were repeated three times and then hASCs were used for subsequent experiments [12].

### 4.3. Extraction and Dedifferentiation of Mature Adipocytes from Adipose Tissue (DFAT)

The procedure for isolation of mature adipocytes derived from white fat tissue was applied with a modification of the method described previously [12,28]. Briefly, after collagenase II treatment (see previous paragraph) the cell suspension was centrifuged at 1100 rpm for 3 min. The floating top layer containing unilocular adipocytes was collected and washed with GM. After two washings, cells (2 × 10^3^/cm^2^) were placed in culture flasks completely filled with DMEM F12-HAM supplemented with 20% FBS. The flasks were turned and incubated in a humidified atmosphere of 95% air with 5% CO_2_ at 37 °C for one week. Cells floated up and adhered to the inner ceiling of the flask; after one week the medium was removed and the flask was turned 180° so that the cells would now sit on the bottom. Subsequently, the cells were cultured in GM and expanded similarly to the hASC.

### 4.4. Flow Cytometric Analysis

Both hASCs and DFAT, at passage 0, were suspended in a phosphate buffer solution (PBS) (Sigma-Aldrich) with 5% human serum (Catalog Number S4200, Biowest, Nuaill, France) at a density of 10 × 10^5^ per tube. The cells were immunostained with anti-human primary antibodies (mAbs) conjugated with fluorescein isothiocyanate (FITC) or phycoerythrin (PE) for 20 min at 4 °C in the dark. The following mAbs were used: anti-CD13 PE, anti-CD14 FITC, anti-CD34 FITC, anti-CD45 PE, anti-CD73 PE, anti-CD90 PE, anti-CD105 PE (Beckman Coulter, Milan, Italy). After washing in PBS with 5% human serum, the cells were centrifuged at 1300 rpm for 1 min. The cells were analyzed with a Navios flow cytometer (Beckman Coulter). Data were acquired, displayed and elaborated by Kaluza 1.2 software package (Beckman Coulter) and the positive cells were counted and compared with the signal of corresponding immunoglobulin isotypes.

### 4.5. Curves of Viability

3 × 10^3^ hASC and DFAT were seeded in 12 well format plates (Sigma-Aldrich) in GM. Every 24 h for one week; cells were trypsinized with Trypsin EDTA (Biowest), centrifuged at 1200 rpm for 10 min at 22 °C, suspended in GM and counted with Burker’s chamber after Trypan Blue (Biological Industries, Cromwell, CT, USA) staining at a dilution 1:1 with PBS. The microscopic observation allowed us to perform a quantitative analysis: the number of viable cells per milliliter was expressed as the difference between the total cell count and the number of non-viable cells per milliliter. The viable cell count was expressed as the mean of two independent counts. 

Cell generation time (g) was calculated by the function: g = *t*/*n* where: *t* = 1; *n* = (Log N−Log N0)/(Log 2); N = cells number; N0 = initial cell number.

### 4.6. Cell Seeding, Culture, and Differentiation

At the third passage of cells culture, the cells (both hASC and DFAT) were seeded at 2 × 10^3^/cm^2^ concentration in 35-mm dishes and grown to semi-confluence in GM.

For osteogenic differentiation, the cells were cultured for seven, 14, and 21 days in DMEM F12-HAM containing 15% FBS, 10 mM betaglycerophosphate, 100 nM dexamethasone, 0.05 mM ascorbic acid (all chemicals from Sigma-Aldrich), 1% antibiotics (100 U/mL penicillin, 100 µg/mL streptomycin), and 1% antifungal (0.25 µg/mL amphotericin) (osteogenic medium, OM).

For adipogenic differentiation, the cells were cultured for three days in DMEM F12-HAM containing 10% FBS, 100 nM dexamethasone, 100 µM insulin, 100 µM rosiglitazone, 250 µM isobutylmethylxanthine (all from Sigma-Aldrich) 1% antibiotics, and 1% antifungal (adipogenic inducing medium, AM). Subsequently, the medium was substituted with DMEM F12-HAM containing 10% FBS, 100 nM dexamethasone, 100 µM insulin, 1% antibiotics, and 1% antifungal (maintenance medium) for 12 days. 

For chondrogenic differentiation, the cells were cultured for 30 days in DMEM F12-HAM containing 1% FBS, 100 nM dexamethasone, 0.05 mM ascorbic acid, 10 ng/mL transforming growth factor-β1 (TGF-β1), 1× insulin-transferrin-sodium selenite (ITS) (Sigma-Aldrich), 1% antibiotics and 1% antifungal (chondrogenic medium, CM). For each differentiation condition, control cells were cultured in GM.

### 4.7. Cell Differentiation Analysis

Osteogenic differentiation—Alkaline phosphatase activity (ALP) was evaluated with a histoenzymatic stain reaction (Bio-Optica, Milan, Italy), following the manufacturer’s protocol. The cells were treated with the solution alfa naphthyl phosphate 15 mg + fast blue RR 10 mg + borate buffer pH 8.8, and incubated at 37 °C for 1 h at room temperature. After washing with distilled water, the cells were fixed with 10% formalin at room temperature for 10 min and then washed with distilled water again. The stained wells were observed with a Leica DM IL microscope (Leica Microsystems Srl, Buccinasco, Milan, Italy). In addition, ALP activity was measured according to Murer et al. [43]. After rinsing in PBS, the cells were incubated with 11 mM p-nitrophenyl-phosphate in 50 mM glycine, 1 mM MgSO4, 1 mM ZnSO_4_, pH 10.5 at 37 °C for 30 min at room temperature, then the reaction was stopped with 1 M NaOH. The absorbance of the p-nitrophenol formed was read at 410 nm with Nanodrop™ system (Nanodrop Technologies, Wilmington, DE, USA). The data are expressed as nmol p-nitrophenol/mg protein × min. Protein content was determined using the Lowry method [44].

Calcium deposits in osteogenic differentiated hASC and DFAT were evaluated using Alizarin Red S staining (IHC World, Woodstock, MD, USA). Each well was fixed with 10% formaldehyde for 15 min at room temperature, rinsed with PBS, added with 40 mM Alizarin Red S, pH 4.1–4.3 for 20 min at room temperature, with gentle agitation, and finally the stained wells were observed with Leica DM IL microscope. Subsequently the stain was eluted by adding 1 mL 10% cetylpyridinium chloride (CPC) (Sigma-Aldrich) for 1 h at room temperature with gentle agitation. Subsequently the absorbance was measured with the Nanodrop™ system at 562 nm.

Adipogenic differentiation—Lipid droplets staining was performed with a modified Oil red O (Bio-Optica) procedure. The cells were rinsed with PBS and incubated for 20 min at room temperature in Oil red O solution (3 mg/mL in 100% iso-propanol). After washing with 60% isopropanol for two seconds to remove excess stain, the cells were washed with PBS and observed under a Leica DM IL microscope. The densitometric analysis and quantification of lipidic droplets were performed on digitized images of Oil Red O staining (magnification 10×) using ImageMaster TotalLab v 1.00 software (Amersham Pharmacia Biotech, Cologno Monzese, MI, Italy). Data are presented as arbitrary units.

Chondrogenic differentiation—Extracellular matrix protein accumulation was measured by staining with 1% Alcian blue 8GX in 3% acetic acid, pH 2.5 (Bio-Optica). Following fixation with 4% paraformaldehyde for 20 min at room temperature and washing with PBS, the cells were treated with 10 drops of Alcian blue 8GX solution for 30 min. After removing, the staining solution, the cells were treated with 10 drops of sodium tetraborate hydroalcoholic solution for 10 min and then washed with distilled water. Finally, each well was observed under a Leica DM IL microscope.

### 4.8. RNA Isolation and Reverse Transcriptase Quantitative Real-Time PCR

Total mRNA of both hASC and DFAT was extracted with QIAzol Lysis Reagent (Qiagen, Milan, Italy) to evaluate gene expression. The monolayers were rinsed twice with PBS and then β-mercaptoethanol (Sigma-Aldrich), and lysis buffer was added. The suspension was frozen in liquid nitrogen and stored at −80 °C until extraction. The degree of purity of RNA was increased by adding 70% ethanol and DNAse (Qiagen). The total RNA extracted was reverse-transcribed into cDNA using random hexamers and M-MLV Reverse Transcriptase (Promega, Madison, WI, USA), according to Laforenza et al. [45]. The mRNA of control cells was extracted from hASC and DFAT maintained in GM. 

Quantitative real-time RT-PCR was performed in triplicate using 1 μL cDNA, obtained as above, using specific primers: *ALP* (Carlo Erba reagents Dasit Group, Cornaredo, Italy) and *RUNX2* (Invitrogen, Carlsbad, CA, USA) (osteogenic differentiation), *LPL* (Carlo Erba reagents Dasit Group), *GLUT4* (Carlo Erba reagents Dasit Group), *adiponectin* (Carlo Erba reagents Dasit Group) (adipogenic differentiation) (Table 3), *ACAN* (Quantitect Primer Assay QT 00001365, Qiagen), *SOX9* (Quantitect Primer Assay QT 00001498, Qiagen) (chondrogenic differentiation). Quantifast-SYBRV^®^ Green PCR Kit (Qiagen) was used in according to the manufacturer’s instruction and qPCR was performed using Rotor Gene 6000 (Corbett). PCR cycles are listed in Table 4. 

Melting curves were generated to identify the melting temperatures of specific products after the PCR run. The qPCR reactions were normalized to the expression of the housekeeping gene *GUSB* (β-glucoronidase, a hydrolase that degrades glycosaminoglycans, including heparansulfate, dermatansulfate and chondroitin-4,6-sulfate) (Hs_GUSB_1_SG, Quantitect Primer Assay QT00046046, Qiagen, Hilden, Germany). Relative gene expression of hASC and DFAT were compared to the relative gene expression of hASC e DFAT in growth medium (control) (Δ*C*t). Fold change values were expressed as 2^−ΔΔ*C*t^.

### 4.9. Statistical Analysis

Results were expressed as the mean ± error standard (ES) of 12 different cell samples; each assay was performed in triplicate.

Statistical analyses were performed using Student’s *t*-test or the one-way ANOVA method followed by the Newman–Keuls’s *Q* test (GraphPad Prism 4.00, 2003).

Statistical significance was established at *p* ≤ 0.05.

## 5. Conclusions

In conclusion, both the hASC and DFAT showed similar morphological characteristics, growth rate, generation time, and surface marker expression. Both populations responded to specific differentiation factors for mesodermal cell lines, thus being able to differentiate into osteogenic, adipogenic, and chondrogenic lineages, as evidenced by the positivity of all parameters. It is suggested that these cell populations may represent an ideal source of cells for regenerative medicine and tissue engineering. In orthopedic as well as maxillofacial applications, they would allow us to regenerate damaged tissue and thus restore the function lost after disease, trauma, or malformation. The cell type preferred for clinical application depends on the tissue to be repaired. For adipose tissue injury, DFAT is preferred due to their commitment to the adipocyte line. 

Before clinical application is possible, some fundamental aspects need to be addressed: a distinct cell surface antigenic profile allowing us to distinguish them from other stem cell populations, their chromosomal stability and the absence of mutations, and culture conditions that might influence the dedifferentiation process.

## Figures and Tables

**Table 1:** Phenotypic analysis by flow-cytometry of human adipose stem cells (hASC) and dedifferentiated mature adipocyte (DFAT). Percentage data of the expression of cell-surface antigens in hASC and dedifferentiated adipocytes at passage 0. CD13, CD73, CD90, and CD105 as a typical panel of mesenchymal stem cells; CD14, CD34, and CD45 as hematopoietic antigens. Data are representative of 12 subjects.

Antigen	hASC	DFAT
CD14	0%	0%
CD34	0%	0.5%
CD45	0%	0.2%
CD13	99.5%	99.2%
CD73	97.9%	95.0%
CD90	99.5%	99.0%
CD105	99.3%	99.1%

**Table 2:** Quantification of lipid droplets in hASC and DFAT on digitized images of Oil Red O staining at 15 days. *, *p* ≤ 0.05.

Quantification of Lipidic Droplets (Arbitrary Units ± S.E.)	
hASC	2,500,000 * ± 711,000
DFAT	32,900,000 ± 3,800,000

**Table 3:** Specific primers for *ALP*, *RUNX2*, *LPL, GLUT4*, and *adiponectin.*

Specific Primers		
**Gene**	**Sense Primers**	**Antisense Primers**
*ALP*	5′-AGCCCTTCACTGCCATCCTGT-3′	5′-ATTCTCTCGTTCACCGCCCAC-3′
*RUNX2*	5′-GGACGAGGCAAGAGTTTCAC-3′	5′-TGCCTGCCTGGGGTCTGTAA-3′
*LPL*	5′-CTGGACGGTAACAGGAATGT-3′	5′-TCCTCCTCCATCCAGTTGAT-3′
*GLUT4*	5′-ATAGGCTCCGAAGATGGGGAA-3′	5′-AAACTGCAGGGAGCCAAGCA-3′
*Adiponectin*	5′-GGAGATCCAGGTCTTATTGG-3′	5′-ACTGAATGCTGAGCGGTATA-3′

**Table 4:** Cycling conditions for osteogenic, adipogenic, and chondrogenic differentiation. (min, minutes and s, seconds).

Cycling Conditions				
Lineage	Initial Denaturation and Time	Cycles of Denaturation, Temperature and Time	Annealing Temperature and Time	Elongation Time
Osteogenic differentiation	95 °C—5 min	40 cycles—95 °C—30 s	58 °C—30 s	72 °C—40 s
Adipogenic differentiation	95 °C—5 min	40 cycles—95 °C—15 s	61 °C—30 s	72 °C—45 s
Chondrogenic differentiation	95 °C—5 min	40 cycles—95 °C—30 s	61 °C—30 s	72 °C—45 s
